# Matrix analyses of pharmaceutical products for the years 2017 to 2019 among public health facilities in Hadiya zone, Ethiopia: a cross-sectional descriptive study

**DOI:** 10.1186/s12913-022-07568-4

**Published:** 2022-02-07

**Authors:** Tamirat Yohannes, Bekele Boche, Nimona Birhanu, Tadesse Gudeta

**Affiliations:** 1 Department of Pharmacy, College of Medicine and Health Sciences, Wachamo University, Hosaena, Ethiopia; 2 Department of Social and Administrative Pharmacy, School of Pharmacy, Institute of Health, Jimma University, Jimma, Ethiopia

**Keywords:** Matrix analyses, Pharmaceuticals, Public health facilities, Ethiopia

## Abstract

**Background:**

To date, global healthcare spending becomes a primary concern, and pharmaceutical costs are the main drivers. The issue is more pressing in developing countries like Ethiopia. However, there is a scantiness of comprehensive data on inventory control practices in health facilities. This study, therefore, aimed to assess the criticality, financial value, and consumption patterns of pharmaceuticals using inventory matrix analyses and explore the related challenges.

**Methods:**

A cross-sectional study supplemented with qualitative assessments was carried out from December 2020 to January 2021 in public health facilities. Three hospitals and 14 health centers were proportionally selected using a simple random sampling technique. Self-administered questionnaires and review of logistics documents and databases like Dagu-Facility were used to obtain the quantitative data. The data were analyzed using excel spreadsheets and SPSS version 23. We gathered the qualitative data through face-to-face in-depth interviews.

**Results:**

The facilities spent 66,312,277.0 Ethiopian birrs to procure 518 pharmaceuticals between 2017 and 2019. Of the total products, 68 (13.1%) belonged to class A and 353 (68.1%) belonged to class C. Among 427 items identified by VEN analysis, 202 (47.3%) were vitals, and 201 (47.1%) were essential products making the highest proportions. Cross-tabulations of ABC and VEN showed that 230 (53.9%) items formed category I, representing 84.3% of total expenditures. Sterile surgical gloves #7.5, amoxicillin capsules, examination gloves, and 40% dextrose injection were among the top-ten high-value closing inventories, accounting for 21% of class X items. The fast-moving items were the most prevalent in all years, accounting for more than 45%, and shared the maximum expenditure, up to 90%. Scarcity of infrastructure and skilled human resources, shortage of pharmaceuticals and problems with suppliers, and management issues were the major challenges in the health facilities.

**Conclusion:**

Most of the items identified by ABC-VEN and FSN-XYZ were Category one, i.e., mainly vital costly products and a few fast-moving items with high closing inventory values, respectively, suggesting close supervision. However, several issues became impediments. Hence, facilities should alleviate the bottlenecks and monitor the stock status to prevent theft and stock out.

## Background

Pharmaceuticals are essential components of health care systems, and at the same time, they share substantial health care expenses. The high percentage of medicines spending, especially in countries with limited resources, is paid out of individuals' pockets. This imposes financial burdens on patients and creating an additional problem on policymakers [1, 2]. On the other hand, health is a fundamental human right that can also be realized through access to essential pharmaceuticals [3]. However, one-third of the world's population lacks an opportunity to obtain essential medicines, and it is more severe in low- and middle-income countries [4], causing patients to suffer from even minor illnesses [5]. From the systematic review by Tewuhibo D et al., in Ethiopia, the average availability of essential drugs from 2003 to 2019 did not exceed 75% [6].

In this sense, the assessment of supply chain systems, including inventory control, is crucial to discern current expenditures for pharmaceutical products and patterns of consumption to design effective policies aimed at cinching universal access to essential medicines. An Effective Supply Chain (ESC) ensures the sustainable availability of quality, safe, and effective pharmaceutical products [7, 8]. ESC can be achieved when proper pharmaceutical selection, quantification, procurement, and use are carried out taking into account consumption rates, clinical significance, and product costs [1, 7, 9].

To this end, several inventory control mechanisms exist to assess the clinical and financial implications of pharmaceutical consumption patterns [10–12]. These include; ABC analysis (always better control); This technique helps classify pharmaceutical items based on their financial value into A (few high priced items), B (medium level of moderately priced items), and C (a large number of low priced items).VEN analysis; classifies pharmaceutical products according to their criticality for health services and the prevention of death or disability into Vital, Essential, and Non-Essential. The other techniques are FSN and XYZ analyses which classify inventory items based on their consumption rates (fast, slow, and non-moving) and closing inventory values (XYZ) [10, 12]. Nonetheless, each technique has its limitations, and thus the combination of them helps to take advantage of the specific techniques [10]. ABC-VEN matrix gives results based on the economic as well as the critical value of pharmaceuticals concurrently [13]. This matrix is a strong tool for a critical appraisal of pharmaceutical product use and assists in containing the value for essential medicine by permitting the expenditure on indispensable items [14]. Cross-tabulating XYZ and FSN provides insight for pharmaceutical usage or patterns of movement and their closing inventory values [15].

However, the poor inventory control system in health care institutions in developing countries, including Ethiopia, becomes a driver for insufficient or surplus pharmaceuticals, leading to depletion or expiration of stocks and inappropriate budget expenditure [16–18]. The study conducted in Kenya revealed that there was a huge mismatch between the criticality of the product (VEN) and expenditure-based classification (ABC) of pharmaceutical items [19]. FSN–XYZ matrix analysis done in Ethiopia showed that an unexpected amount of budget (20%) was disbursed on high-cost and non-moving pharmaceutical items [20]. Moreover, the study conducted in northern Ethiopia showed that a significant amount of the annual budget (75.86%) was spent on very few pharmaceutical products (17%), which needs management review and engagement in the whole process of the hospital's supply chain decisions [21].

Though few studies were conducted on inventory control practice using matrix analyses in some regions of Ethiopia, almost all have limitations. Most of them addressed ABC-VEN alone in the same types of health institutions, e.g., [7, 21–23]. The others studied at a supplier agency [11] or in a single zone [20]. Yet all of them lacked triangulation with qualitative data to provide a deep and complete understanding of the phenomenon. Furthermore, despite differences in regional administration, no study has been conducted in the Hadiya zone of the Southern Nations, Nationalities, and Peoples' Region (SNNPR). This study evaluated pharmaceutical inventories in hospitals and health centers using comprehensive techniques and qualitative data as a supplement. Thus, besides the clinical importance and drug expenditure, it will add information, such as the closing inventory values of pharmaceuticals with various consumption rates and associated bottlenecks to the existing body of literature. Hence, this study aimed to assess the criticality, financial value, and consumption patterns of pharmaceuticals using inventory matrix analyses techniques (ABC-VEN and FSN-XYZ) and also explore challenges in public health facilities of the Hadiya zone Ethiopia, with the goal of better managerial actions.

## Methods

### Study area and period

The study was conducted from December 15, 2020, to January 15, 2021, in selected public health facilities in the Hadiya zone, the SNNPR, Ethiopia. The zone covers an area of 3542.66 square kilometers and accounts for 3.8% of the total area of the region. It has 17 administrative districts. According to the Ethiopian Central Statistical Agency of 2007 last report, the population of the Hadiya zone was 1,243,776 [24]. Access to healthcare in the zone is ensured through public and private health care systems. Currently, there are 376 public health facilities, including four hospitals, 61 health centers, and 311 health posts, in the zone. A total of 1918 healthcare professionals from diverse backgrounds serve the facilities. These include 135 medical doctors, 95 health officers, 495 nurses, 96 midwives, 81 pharmacists, 218 laboratory specialists, 131 druggists, 629 health extension workers, 28 environmental health specialists, seven anesthesiologists, and three biomedical engineers.

### Study design

A cross-sectional study supplemented with qualitative data was conducted to evaluate and explore pharmaceuticals inventory control and underlying challenges in healthcare facilities. The qualitative data were used in the discussion part to complement the quantitative findings.

### Source and study population

The source population comprised all health facilities in the Hadiya zone, pharmaceuticals, health professionals working in those health facilities, good reception and issuance vouchers Models, records of closing inventory, and a local database, i.e., Health Commodity Management Information System (HCMIS) or the Dagu Facility [25]. The researchers used models 22, HCMIS, documents of closing inventory, and health professionals to source the intended data. The study populations were selected public hospitals and health centers, and pharmaceuticals managed and purchased by the facilities from 2017 to 2019.

Public hospitals and health centers that started operating three years ahead of the data collection period were included. Under Ethiopia's current health care structure, health posts act as dispensaries and are supplied directly by health centers [26]. Therefore, we excluded them from the study. Health professionals with service years of greater than three years in the health facility were recruited in both the quantitative and qualitative study. All drugs labeled as program commodities [27], including antiretroviral, anti-TB drugs, malarial commodities, vaccines, and family planning products were also left out from this study. In Ethiopia, these products are purchased at the central level by Ethiopian Pharmaceuticals Supply Agency (EPSA). As medical pieces of equipment are durables, we omitted them from the list.

### Sampling procedures of health facilities

First, the Hadiya zone was randomly selected from 15 zones in the SNNPR [28] using a lottery method. Then the health facilities (HFs) were selected from the area. We used the USAID delivery project recommendation [29] to determine the sample size of the HFs. It recommends taking at least 15% of the total facilities to increase the power of generalization. Accordingly, the calculation gives about 10 health facilities taking the current total number of public hospitals and health centers (65 HFs) into account. However, to address all the 17 districts of the zone, we planned to randomly select one facility from each district. Three out of four hospitals were chosen considering the service year. The health centers were chosen from the districts where no hospitals were selected. Pharmacy heads and store managers were considered experts in this area and thus participated in qualitative data collection. In general, 19 participants (15 males and 4 females) were involved, and the sample size depended on the information saturation, i.e., the interviews ceased when similar issues seem to be repeated.

### Data collection procedures

Pretested data extraction formats, developed based on previous literatures, were used to collect the desired information. The facility and participants' profile data were obtained from store managers and pharmacy heads available during the visit and volunteered to participate in the study. The annual consumptions and the respective value of each pharmaceutical product for three consecutive years, September 2017 to September 2019, were used in the ABCanalysis. These data were obtained by reviewing issuance documents (Model 22) used during the specified years. The researchers also extracted the data from Dagu-facility (HCMIS) software for facilities using the program. The clinical staff and standard treatment guidelines were consulted to determine the VEN category. The data for the XYZ analysis were obtained by reviewing the three-year closing inventory files and the facilities' HCMIS. The Fast, Slow, and Nonmoving (FSN) items identification was undertaken based on the frequency of pharmaceutical issuance per annum from the main store to different departments within the HFs. The data for this analysis were acquired from Model 22 and electronic record (HCMIS). The qualitative data were collected through face-to-face in-depth interviews. The questions were comprehensive and probing types. To ensure consistency throughout the interviews, the principal investigator moderated the discussion. Each interview lasted an average of 20 min and was audio-recorded using a smartphone. A local language, Amharic, was used at the interviewees' convenience.

### Data analysis

SPSS was used to analyze socio-demographic and facility-related variables, and the ABC, VEN, XYZ, and FSN matrixes were analyzed using Microsoft Excel. Below are brief descriptions of the procedures for the matrix analyses.

### ABC analysis

The total quantities of each pharmaceutical issued in the last three years, from September 2017 to September 2019, were compiled. The annual monetary value of each product was computed by multiplying a unit price by the quantity of each product. The percentage of value attributed to specific pharmaceuticals was determined and arranged in descending order. The next step was calculating the cumulative percentage for a discrete item and classifying them into A, B, and C classes based on the following thresholds. A class: about 10% of the items account for around 70% of the total values, B class: Consist nearly 20% of the items and takes roughly 20% of the total annual expenditure, and C class: includes around 70% of the items representing only 10% of the total annual value [9].

### VEN analysis

Based on the clinicians' grading and treatment guidelines, the VEN analysis looked at the health impact of pharmaceuticals in the health facilities. The products used to prevent or treat serious illnesses and couldn't be substituted for other products were considered vital (V). To ensure consistency in reporting, items identified as vital in a particular institution remained the same in all facilities, even if they were essential in other settings. Pharmaceuticals somehow substituted and used against less severe but significant illnesses were classified under essential (E) items. Products indispensable for healthcare provision, but their absence didn't interrupt the services were regarded as non-essential (N) products [30].

### ABC-VEN matrix analysis

we cross-tabulated the ABC and VEN results to generate different couples and groups of the pharmaceuticals. Accordingly, nine pairs of items were formed and divided into three categories. The first category comprised mainly the expensive and vital products (AV + AE + AN + BV + CV), the second category included most of the less costly and essential items (BE + BN + CE), and the third category contained the cheapest non-essential products (CN) [9, 13].

### XYZ analysis

The closing inventory values for each fiscal year, 2017–2019, were sorted and arranged in descending order, and the cross-ponding cumulative percentage was computed. The items were then grouped into the X, Y, and Z classes based on the following cutoff points; the first 70% of the total inventory value corresponds to X class, the next 20% are of Y class, and the last 10% of the value corresponds to the Z class [10].

### FSN analysis

There is no common guiding principle for classifying commodities as fast-moving (F), slow-moving (S), and non-moving (N) [31]. However, for this particular study, we arranged pharmaceuticals procured from 2017–2019 in descending order based on their average frequency of issuance in a specific year. The resulting values were used to classify the items based on thresholds stipulated in previous studies. Ultimately, the products issued on average ≥ 15, 5–15, and < 5 times comprised F, S, and N items, respectively [20, 31].

### FSN-XYZ matrix analysis

The FSN and XYZ items coupling was done through a cross-tabulation on an excel spreadsheet. Nine couples were formed and then classified under three categories. Category I commonly comprised the fast and high-value items, including FX, FY, FZ, SX, and NX. The second category constituted SY, SZ, and the last contained the nonmoving and low-value items (NZ). At the last, the quantitative findings were summarized using tables and figures [10].

A thematic analysis technique was used to analyze the qualitative results. The records were transcribed to the English language by the authors and verified by an expert from Jimma University. After the rehearsal of the texts, variables were coded manually in a word document to identify appropriate themes. The themes were described in narrative form, followed by quoting the opinions of some respondents. Finally, the findings were used at the discussion phase to support the quantitative results.

### Data quality assurance

We trained quantitative data collectors for one hour on the data collection process and how to acquire the intended information. And the investigators oversaw their daily activities by checking the completeness of the data extraction formats. The formats were developed by examining various types of literature and were evaluated by relevant and experienced researchers to maintain content validity. Moreover, a pre-test was conducted in two randomly selected health facilities in the Hadiya Zone, where both were excluded from the actual study. After the test, we reviewed each questionnaire for changes in responses for different administrations under similar circumstances and determined the variations. All questions produced consistent results and, therefore, were reliable. Concerning the qualitative assessment, all researchers participated in the transcription and compilation of the data. The researchers have experience in qualitative and quantitative research and are all pharmacists with master's degrees in pharmaceutical supply chain management. Besides, a qualitative research expert was also involved in verifying the transcription.

## Results

### Characteristics of respondents and health facilities

We visited fourteen health centers and three hospitals, and all of them were volunteers making a response rate of 100%. The self-administered questionnaire showed that only 5 out of 17 health institutions had a pharmacy manager (three hospitals and two health centers) at the time of the visit. Out of the total facilities, only 2 (11.8%) and 6 (35.3%) of them had electronic records and functional DTC, respectively. Eight (47.1%) facilities had drug formulary or standard treatment guidelines. Only 4(23.5%) of the facilities got adequate support from the top-level management. In terms of human resources, the health facilities had 89 employees under the pharmacy department serving in various positions such as dispensaries, store manager, and pharmacy headship. Pharmacy heads (five) and store managers (eighteen) participated in the study. Of the participants, 20 (87.0%) were pharmacy professionals, and 17 (73.9%) had diplomas. Twelve of them (52.2%) had more than five years of work experience. Sixteen (69.6%) respondents received IPLS training. Only nine respondents (39.1%) were satisfied with their current job (Table 1).

**Table 1:** Characteristics of health facilities and respondents in public health facilities of Hadiya zone, Ethiopia

Variables		H n (%)	HCs n (%)	Total n (%)
**Characteristics of respondents per health facilities**				
Qualification	Degree	5(21.7)	1(4.4)	6(26.1)
	Diploma	2(8.7)	15(65.2)	17(73.9)
Profession	Pharmacy	7(30.4)	13(56.5)	20(87.0)
	Nursing	0	3(13.0)	3(13.0)
Work experiences	1–5 years	4(17.4)	7(30.4)	11(47.8)
	> 5 years	3(13.0)	9(39.1)	12(52.2)
Type of training received	IPLS^a^	4(17.4)	12(52.2)	16(69.6)
	Both LMIS^b^ and APTS^c^	3(13.0)	1(4.4)	4(17.4)
	Not trained	0	3(13.0)	3(13.0)
Working position	Pharmacy head	3(13.0)	2(8.7)	5(21.7)
	Store manager	4(17.4)	14(60.9)	18(78.3)
Job satisfaction	Satisfied	5(21.7)	4(17.4)	9(39.1)
	Not satisfied	2(8.7)	12(52.2)	14(60.9)
**Characteristics of health facilities**				
Electronic records	Available	1(5.9)	1(5.9)	2(11.8)
	Not Available	2(11.8)	13(76.5)	15(88.2)
Functional DTC^d^	Available	2(11.8)	4(23.5)	6(35.3)
	Not available	1(5.9)	10(58.8)	11(64.7)
DF^e^ or standard treatment guidelines	Available	3(17.7)	5(29.4)	8(47.1)
	Not available	0	9(52.9)	9(52.9)
Support from top-level management	Adequate	1(5.9)	3(17.7)	4(23.5)
	Not adequate	2(11.8)	11(64.7)	13(76.5)
Number of staff under pharmacy unit	**–**	53(59.6)	36(40.5)	89(100)

^a^Integrated pharmaceutical logistics service, ^b^logistics management information system, ^c^auditable pharmaceuticals transaction system, ^d^drug therapeutics committee, ^e^Drug formulary

### ABC analysis

**Fig. 1:**
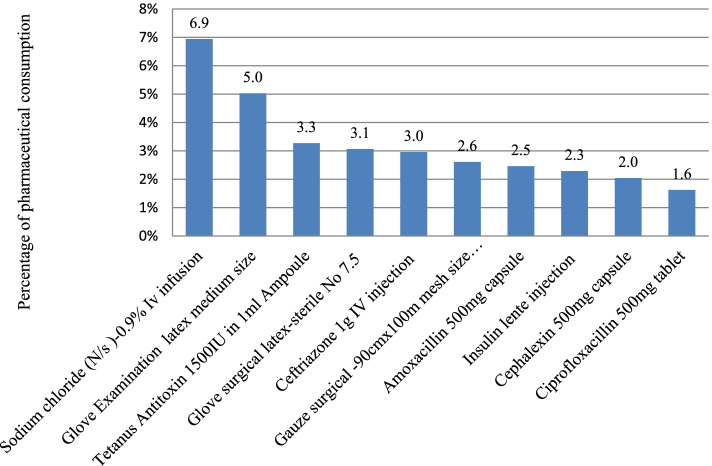
Top ten most costly pharmaceuticals in selected health facilities of Hadiya zone, Ethiopia

Ethiopian Birr (ETB) 66,312,277.0 spent on 518 items between 2017 and 2019 in the health institutions of the Hadiya zone. Of the total items consumed in three consecutive years, 68 products (13.1%) belonged to class A, accounting for ETB 46,556,646.2 (70.2%). B class contained 97(18.7%) items taking ETB 13,417,858.3 (20.2%) of the total pharmaceuticals expenditure. Three hundred fifty-three (68.1%) products with a value of ETB 6,337,772.4 constituted C class items (Table 2). Out of the 68 class A items, Sodium chloride(N/S)-0.9% IV infusion, Glove examination latex medium size, and Tetanus Antitoxin 1500 IU in 1 ml Ampoule contributed 15.2% of the total expenditures (Fig. 1).

**Table 2:** ABC and VEN analysis of pharmaceuticals in selected health centers and hospitals in the Hadiya zone, Ethiopia

ABC	Health centers			Hospitals			Aggregate		
	Number of items (%)	Consumption value in ETB	Value in %	Number of items (%)	Consumption value in ETB	Value in %	Number of items (%)	Consumption value in ETB	Value in %
A	31(15.4)	10,591,336.1	70.0	66(13.3)	36,022,489.3	70.4	68(13.1)	46,556,646.2	70.2
B	46(22.8)	3,093,675.1	20.4	90(18.2)	10,281,605.8	20.1	97(18.7)	13,417,858.3	20.2
C	125(61.9)	1,449,581.8	9.6	341(68.9)	4,873,588.9	9.5	353(68.1)	6,337,772.4	9.6
Total	202(100)	15,134,593.0	100%	495(100)	51,177,684.0	100	518(100)	66,312,277.0	100
VEN									
V	58(37.4)	5,656,545.2	39.2	167(40.8)	28,937,902.4	58.8	202(47.3)	41,045,181.4	64.0
E	79(51.0)	8,320,401.6	57.7	220(53.8)	18,934,672.0	38.5	201(47.1)	21,725,445.0	33.9
N	18(11.7)	446,317.0	3.1	22(5.4)	1,324,209.5	2.7	24(5.6)	1,338,161.4	2.1
Total	155(100)	14,423,263.8	100	409(100)	49,196,783.9	100	427(100)	64,108,787.8	100

### VEN analysis

Of the items identified through VEN analysis (*n* = 427), 202(47.3%) of them were vitals accounting for 64% of the drug costs. Two hundred one (47.1%) of the pharmaceuticals belonged to essential items and they took about 33.9% of the total annual budget. There was a slight variation in the proportion of vital items in hospitals (40.8%) and health centers (37.4%). However, non-essential items were more prevalent in health centers than hospitals accounting for 11.7% and 5.4% of the total products, respectively (Table 2).

### ABC-VEN coupling

Matrix analysis of 427 medicines based on the ABC-VEN combination showed that 230 (53.9%) items included in category I (AV + AE + AN + BV + CV) accounting ETB 54,053,951.1 (84.3%) of total pharmaceuticals spending (TPS) (Table 3). Only 21(4.9%) products formed category III (CN) accounting for 193,465.4 (0.3%) the TPS. However, the individual couples indicated that 124 (29%) of the products were less costly vital (CV) items with an annual consumption value of ETB 2,796,654.9 (4.4%). The vital expensive products (AV) contained 39(9.1%) items taking more than half, ETB 33,185,119.6 (51.8%), of the allocated budget. Substantial percentage variation with category II was observed between hospitals, 190(46.5), and health centers, 60 (38.7%) (Table 3).

**Table 3:** Cross-tabulation of ABC and VEN pharmaceuticals in selected health centers and hospitals in Hadiya zone, Ethiopia

ABC-VEN category	Health centers			Hospitals			Aggregate		
	No (%)	Consumption in ETB	%age^a^	No (%)	Consumption in ETB	%age	No (%)	Consumption in (ETB)	%age
AV	9(5.8)	4,412,094.4	30.6	27(6.6)	23,515,608.8	47.8	39(9.1)	33,185,119.6	51.8
AE	21(13.5)	5,940,538.6	41.2	32(7.8)	10,567,305.6	21.5	27(6.3)	12,087,220.4	18.9
AN	1(0.7)	238,703.1	1.7	1(0.3)	921,549.3	1.9	1(0.2)	921,549.3	1.4
BV	9(5.8)	586,401.7	4.1	32(7.8)	3,568,151.6	7.3	39(9.1)	5,063,406.9	7.9
CV	40(25.8)	658,049.1	4.6	108(26.4)	1,854,142.1	3.8	124(29.0)	2,796,654.9	4.4
BE	27(17.4)	2,016,597.0	14.0	52(12.7)	6,124,012.9	12.5	51(11.9)	7,251,908.5	11.3
BN	2(1.3)	92,989.5	0.7	2(0.5)	223,146.8	0.5	2(0.5)	223,146.8	0.4
CE	31(20.0)	363,266.0	2.5	136(33.3)	2,243,353.5	4.6	123(28.8)	2,386,316.0	3.7
CN	15(9.7)	114,624.4	0.8	19(4.7)	179,513.5	0.4	21(4.9)	193,465.4	0.3
I	80(51.6)	11,835,786.9	82.1	200(48.9)	40,426,757.2	82.2	230(53.9)	54,053,951.1	84.3
II	60(38.7)	2,472,852.5	17.2	190(46.5)	8,590,513.1	17.5	176(41.2)	9,861,371.3	15.4
III	15(9.7)	114,624.4	0.8	19(4.7)	179,513.5	0.4	21(4.9)	193,465.4	0.3
Total	155(100)	14,423,263.8	100	409(100)	49,196,783.9	100	427(100)	64,108,787.8	100

^a^percentage

### FSN analysis

Of the 518 pharmaceuticals issued in the health institutions, fast-moving items accounted for the highest proportions. Ultimately, in 2017 they attributed to 45.5%, and in 2018 and 2019, they represented 46.5% of the issues. More than 80% of the total expenditure was spent on these products over the three years. On the other hand, slow-moving items were as prevalent as the fast-moving items in the last two years, 2018 and 2019 (Table 4).

**Table 4:** XYZ and FSN classification and cross-tabulation of pharmaceuticals in selected health centers and hospitals in Hadiya zone, Ethiopia

2017					2018			2019		
Class	Frequency	No. of Items (%)	Total value in ETB	%-age^a^	No. of Items (%)	Total value in ETB	%-age	No. of Items (%)	Total value in ETB	%-age
F	> 15	146 (45.5)	16,705,873.4	81.4	168 (46.5)	19,265,277.0	87.1	187 (46.5)	21,328,407.3	90.1
S	5–15	90 (28.0)	2,655,710.8	12.0	167 (46.3)	2,721,620.7	12.3	198 (49.3)	2,248,069.7	9.5
N	< 5	85 (26.5)	1,152,905.6	5.6	26 (7.2)	131,904.6	0.6	17 (4.2)	102,508.0	0.4
Total		321(100)	20,514,489.7	100	361 (100)	22,118,802.3	100	402 (100)	23,678,985.0	100
X		51 (17.3)	11,004,295.8	70.1	64 (23.3)	9,238,916.9	70.3	81 (26.7)	9,448,323.2	70.2
Y		98 (33.2)	3,195,536.1	20.4	95 (34.7)	2,659,802.3	20.2	96 (31.7)	2,720,285.3	20.2
Z		146 (49.5)	1,491,551.2	9.5	115 (42.0)	1,253,713.6	9.5	126 (41.6)	1,288,774.3	9.6
Total		295 (100)	15,691,383.1	100	274 (100)	13,152,432.8	100	303 (100)	13,457,382.8	100
XYZ-FSN coupling										
XF		39 (13.2)	9,409,603.2	60.0	47 (17.2)	7,983,894.4	60.7	62 (20.5)	8,163,335.0	60.7
YF		46 (15.6)	1,518,335.6	9.7	50 (18.2)	1,417,847.7	10.8	50 (16.5)	1,413,860.8	10.5
ZF		31 (10.5)	336,892.9	2.2	36 (13.1)	416,382.3	3.2	34 (11.2)	391,942.1	2.9
XS		9 (3.1)	1,046,564.2	6.7	9 (3.3)	512,304	3.9	10 (3.3)	520,144.7	3.9
XN		5 (1.7)	665,345.7	4.2	9 (3.3)	782,256	5.9	11 (3.6)	845,763.1	6.3
YS		14 (4.7)	423,743.3	2.7	24 (8.8)	664,951.8	5.1	29 (9.6)	774,705.4	5.8
ZS		36 (12.2)	405,155.8	2.6	38 (13.9)	399,560.8	3	46 (15.2)	451,216.1	3.4
YN		36 (12.2)	1,136,239.9	7.2	20 (7.3)	537,465.3	4.1	16 (5.3)	467,036.1	3.5
ZN		79 (26.8)	749,502.5	4.7	41 (14.9)	437,770.5	3.3	45 (14.9)	429,379.4	3.2
Total		295(100)	15,691,383.1	100	274 (100)	13,152,432.8	100	303 (100)	13,457,382.8	100
I		130(44.1)	12,976,741.6	82.8	151(55.1)	11,112,684.4	84.5	167(55.1)	11,335,045.7	84.3
II		86(29.2)	1,965,139.0	12.5	82(30)	1,601,977.9	12.2	91(30.1)	1,692,957.6	12.7
III		79(26.7)	749,502.5	4.7	41(14.9)	437,770.5	3.3	45(14.8)	429,379.4	3.2

^a^percentage

### XYZ analysis

**Fig. 2:**
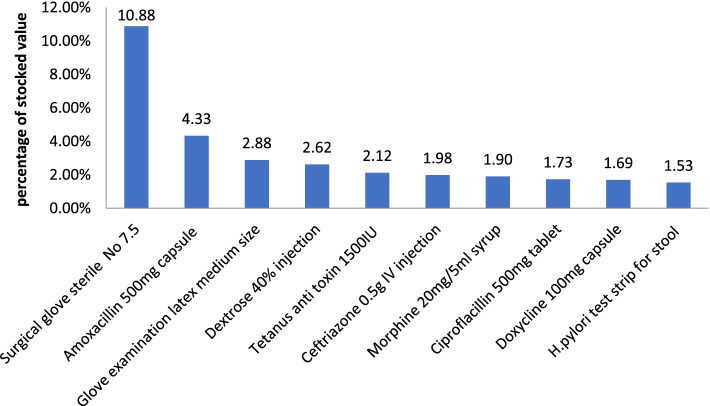
Top ten high value closing inventories at selected health faculties of Hadiya zone, Ethiopia

From the XYZ analysis, most of the closing inventories were Z items comprising about 40%, followed by Y accounting for 30% of the items, and they represented 9.5% to 20.4% of closing stock values (Table 4). The X class had low annual proportions, but it accounted for the highest stock values, around 70% every year. As shown in Fig. 2, the first four mostly stocked high-value items, including sterile surgical gloves # 7.5, Amoxicillin Capsules 500 mg, examination latex gloves medium size, and 40% Dextrose injections, accounted for approximately 21% of X class values (Fig. 2).

### FSN-XYZ coupling

Four hundred thirty-seven kinds of pharmaceuticals were cross-tabulated based on consumption rates and annual closing values. According to the analysis, category one (FX, FY, FZ, SX, and NX) became dominant in the three consecutive years. It accounted for 44.1% in 2017 and 55.1% in 2018 and 2019. It also shared the maximum pharmaceuticals expenditures, up to 84.5%. The XF couple, the expensive fast-moving items, contributed the highest spending, as far as 60%. The proportions of the second (SY and SZ) and the third categories (NZ) didn’t exceed 30% (Table 4).

### Qualitative results

The ages of the participants ranged from 25 to 40 years old, and they had service experiences of 3 to 12 years in their current position. Most of them were pharmacy professionals with a bachelor's degree and a diploma. The interviews also included three clinical nurses from the health centers working as storekeepers. The interviews revealed various bottlenecks in ABC, VEN, XYZ, and FSN inventory control practices in health facilities. The findings were summarized into the following four themes, depending on the characteristics of the data.

### Scarcity of infrastructures

Like human resources, sufficient physical assets are mandatory for effective pharmacy practices, including inventory activities. In contrast, the interviewees revealed that health institutions, especially most health centers, faced shortages of premises, including offices, separate toilets for staff, power outages, and water scarcity. In some institutions, there was difficulty in regular physical inventory counting to identify the status of pharmaceuticals due to narrow and non-standardized storage facilities. Even irrelevant items such as stationary materials and detergents might be stored along with pharmaceuticals in the free spaces in the storerooms. As a result, employees get challenged to sort out, plan, and control their inventories properly and provide data for informed decision-making. The purchasing of pharmaceuticals is mainly carried out based on trends and needs that have emerged from the different departments within the facilities. Only a few facilities had the experience to analyze their products based on their value, importance, and movement for better decisions. One of the interviewees elaborated as follows;

“The issue of physical resources is a big problem in our facilities. For example, I am the head of a pharmacy in this hospital but, I do not have an office. I asked the upper management several times, but there was no response. The hospital management does not have a positive perception of pharmacy services in general. The administration believes that the activity of each pharmacist can take place in a dispensary room. For this reason, we are not motivated to follow inventory control techniques such as ABC, VEN, and ABC-VEN matrix analyses. Therefore, medicines are procured at our facilities through guesswork.” (Male, pharmacy head from a primary hospital).

### Shortage of skilled human resources

The scarcity of qualified and appropriate human resources in almost all institutions became an obstacle to performing pharmaceutical and logistics activities. Most health centers had only two pharmacy experts; one acted as a store manager and the other as a dispenser. Even in three surveyed health facilities, nurses worked on behalf of pharmacy professionals (as pharmacy heads). A store manager at one of the health centers explained the problem as follows;

“It is a good practice to conduct pharmaceutical matrix analyses to improve inventory management. In reality, it is a difficult task in a firm where the scantiness of skilled human resources is a problem. Even, in our facility, non-pharmacy professionals might be assigned to perform dispensing practices during the annual leave, sick leave, or maternity leave of the pharmacists.”(Male store manager, four years of service experience).

Another storekeeper at a hospital added,

''This hospital has various departments, including a Drug Information Center (DIC), compounding unit, community pharmacy, ART pharmacy, OPD pharmacy, inpatient pharmacy, emergency pharmacy, and OBS and GNY pharmacy. Each needs sufficient pharmacy experts. In the actual scenario, only 32 pharmacists provide services in this particular hospital, including the store manager and the head of the pharmacy. Almost all of them are busy dispensing medicines and entering transaction data into a computer program and a model 19. Hence, we are afraid that the additional burdens to the available limited employees will lead to burnout and attritions. For this reason, we seldom practice activities like conducting inventory matrix analyses." (Male hospital store manager, service experience of five years).

### Supplier-related issues and unavailability of pharmaceuticals

The lack of some but critical pharmaceuticals at the Ethiopia Pharmaceutical Supply Agency (EPSA) had led to procurement from private suppliers for costly prices. Even, sometimes they may not be available at private wholesales. On the other hand, EPSA's resistance in supplying the intended types of medicines in the required amount and its pushing practices of irrelevant and or near expiry items to the medical institutions further complicated inventory control systems. A pharmacy head of one health center described;

''When we place orders with the Ethiopian Pharmaceuticals Supply Agency, we should collect surplus products, though they are not part of our demands, to get the intended services. This increases the inventory level that ties up a budget and occupies substantial spaces of the storerooms. The agency also issues near expiry products that may later expire in the health facilities, especially if they are slow-moving items." (Head of the pharmacy, service experience of four years).

Another nurse store manager explained;

“This year, ceftriaxone 1 g and 0.5 g injections have been out of the market for a long time, and now it is bought for 70 and 50 Ethiopian birrs, which is much higher than previous costs, 12.5 and 9.53 birrs, respectively.” (Female store manager, service experience of five years).

### Administration related issues

The negligence, lack of commitment, and attitudes gabs towards pharmaceutical services by senior management and committees in health facilities negatively influenced the morale work of pharmacists. In most of the health institutions, the drug therapeutic committees (DTCs) were not functional for selections and procurements of pharmaceuticals. Physical counts of inventories are hardly conducted regularly, the majority of the facilities counted at the end of the fiscal year. Decisions on pharmaceuticals need the involvement of appropriate professionals. Nevertheless, the discussions and judgments on pharmacy activities in the hospitals and health centers usually ignore relevant professionals. Consequently, the majority of pharmacists and druggists were unsatisfied with their current job and lack the motivation to take additional responsibilities. One of as store managers elucidated the problem as follows,

“The top management of the facility begins to supervise and direct staff to correct naked problems such as organizing shelves and documents when they have an appointment from senior officials to visit and oversee. Otherwise, pharmacy services are of no interest to anyone, and the staff lacks the motivation and morale to carry out their duties.” (Male pharmacy head, five years of service experience).

## Discussion

Inventory control through diversified techniques is a key to the containment of operational costs and the sustainability of pharmaceutical services [32]. A variety of matrix analyses were used in the present study to classify pharmaceuticals in terms of cost, criticality, and stock movements, thereby determining the efficiency of health institutions in budget utilization and inventory management.

ABC analysis revealed that of the 518 pharmaceutical products purchased in the past three years, from 2017 to 2019, 68 (13.1%) belong to class-A and 97 (18.7%) belong to class-B representing ETB 46,556,646.2 (70.2%) and ETB 13,417,858.33 (20.2%) of Total Pharmaceuticals Expenditure (TPE), respectively. Despite the highest proportion of class-C items, the value of class-A products is still high, surpassing three and a half folds the prices of B items. It's an indication of weak inventory monitoring at the facilities, as also evidenced by qualitative data. In reality, class-A items are required in small quantities and need strict control, including frequent counting and better forecasts [33]. Nevertheless, the interviewees revealed that most facilities rarely conducted regular physical inventories due to inadequate workplaces, lack of qualified human resources, and poor administrative support. The results are comparable to reports from Turkey, India, and Arbaminch, Ethiopia [23, 30, 34], but slightly different from the study by Abdelmonim Ahmed H et al. of Sudan [35]. Class B requires moderate regulation, while Class C requires only minimal order and procurement controls, and these actions can be undertaken by middle and lower-level managers, respectively [21]. The share of class-C items in the current study was about 68.14%, which is encouraging.

The present study showed that from 427 pharmaceuticals used in the facilities and identified through VEN analysis, the essential items and vitals took the highest percentages accounting for 47.1% and 47.3%, respectively. Surprisingly, these results are promising despite poor inventory control practices, negative perceptions of facility administrations, human resource constraints, and related issues highlighted in the qualitative data. It's also far better than a report from Sudan and India [35, 36] concerning V items, with only 28%and 33% reports, respectively but comparable in terms of the essentials. It can be a phenomenon associated with procurement based on demand from wards and units of the institutions in the present study. Vital items are lifesaving and cannot be substituted, and require continuous availability and reasonable safety stock. Its absence even for a day is unacceptable and can lead to death and disability of patients and complications of illnesses [37].

The ABC-VEN Matrix dictates the financial and clinical implications of pharmaceutical use. In this study, items classified as Category I (AV + AE + AN + BV + CV) were 230 (53.9%) and consumed 84.3% of the TPE. Category B contained 176 (41.2%) items representing 15.4% of TPE. The less expensive but vitals (CV) and essential items (EC) with percentages of 29% and 28.8%, respectively, were the highest proportions in categories I and II. From the overall findings, category I consumed an enormous percentage of TPE. Hence, more attention and strict management control are required, as these elements are either costly or vital [21]. In contrast, unlike clinical services, the top administration of the facilities had negligence, lack of commitment, and poor perception towards pharmaceutical services, as per information obtained from key informants. These will create a favorable environment for theft and wastage of those expensive or vital products [38, 39].

FSN analysis classifies items based on the rate of consumptions/issuances from the medical store to the various wards, clinics, and units within the facility. It supports timely actions to remove dead stock and prevent accumulation [40]. In the present study, the fast-moving items were the most prevalent in all years, accounting for more than 45%, and shared the maximum TPE, up to 90%. And most of the top ten of these items in the facilities were medical supplies, but health centers also had medicines. Such products need strict monitoring as their absence compromises health care delivery and even leads to dangerous consequences if they are lifesaving ones [40, 41]. The slow-moving items showed an increment in 2018 and 2019, accounting for 46.3% and 49.3% of the issues, respectively. It’s suggestive of a weak monitoring system, for example, less frequency of stock-taking in the facilities, probably because of shortage of skilled human resources, staff dissatisfaction, and scantiness of electronic recording tools. The slow and non-moving items should be at a minimum level to reduce the length of a budget tie-up to products, minimize pharmaceutical waste, and a long-term occupation of a warehouse [38, 42]. The proportion of F items is comparable to a report from West Arsi, Ethiopia (35.3%), and India (46.0%) [20, 43].

XYZ analysis identifies pharmaceuticals that have a high, medium, and low stock value at the end of a fiscal year and helps to determine the amount of budget linked to products at the same time [44]. Class X pharmaceuticals need special attention as they share a high stock value, which can be used for other purposes. Class Y items take an average stock value and require modest control. Substantial portions of products, ranging from 31.7% to 49.5%, in this study were in categories Y and Z, representing a minute fraction of the stock's values. In contrast, a few class-X items, accounting for less than 26.7% of the closing inventories, claimed approximately 71.1% of total stock values on average. The current study facilities outperformed those reported by Reddy DK et al. [7], where only 10% of X items accounted for 59% of total values in tertiary care hospitals in India. The disparity could be due to the variety of items and settings studied, with the latter study focusing on cardiac medicines in a single facility.

According to the FSN-XYZ matrix analysis, category one (FX, FY, FZ, SX, and NX) had the highest proportion in all years, with a minimum of 40.1% in 2017. This class also shared the maximum TPE, up to 84.5% where the FX items contributed the most, about 60%. As these items are expensive and in high demand, stock outs are more likely. According to evidence, more than 80% of Ethiopia's pharmaceutical needs are met through imports from international suppliers, which may result in longer lead times [45]. Thus, the facility administration, EPSA, regional health bureaus, and the Federal Ministry of Health (FMOH) should strengthen the supportive supervision to address the issues such as infrastructure, staff commitment and satisfaction, and administrative supports through diverse platforms and sharing real-time information with various stakeholders, including non-government organizations. The findings are similar to the study by Jobira et al. [20] in health care institutions in the West Arsi region, Ethiopia.

This study had limitations in that a few of the facilities used HCMIS software for information management while the majority used paper-based tools. The data obtained from these two groups of facilities may not be consistent. As some facilities failed to include the price of the pharmaceuticals on their reports (Model 22), we used average prices for the matrix analyses.

## Conclusions

Overall, the results showed that most of the items identified by ABC-VEN and FSN-XYZ were in the first category in both cases, i.e., mainly expensive but life-saving and a few fast-moving with high closing inventory values, respectively. This category could likely be superior, as procurement practice in most facilities was based on clinicians’ suggestions. The pharmaceutical selection and procurement could be improved to avail affordable but still lifesaving items if the facilities had functional DTC and tools like formulary manuals, standard treatment guidelines, and essential medicine lists. Otherwise, it will not be easy to conduct bulk procurement as the prescribers' needs may not be known in advance. However, in the present study, more than half of the institutions lacked the tools and functional DTCs. These items are highly susceptible to theft and depletion and, therefore, require strict controls such as frequent counting, use of lockable cabinets, and, most importantly, automation of inventory control systems. Unfortunately, the current institutions had several problems, including a shortage of qualified personnel, and inadequate electronic registration system, a lack of staff motivation and commitment, and weak administrative support. Therefore, health facilities should work to mitigate the challenges and put in place adequate controls, such as audits, frequent supervision, and maintain low buffer stock to prevent locking up of budget to these items.

## Data Availability

The data sets generated during and/or analyzed during the current study are available from the corresponding author on reasonable request.

## Abbreviations

APTS: Auditable Pharmaceuticals Transaction system
ART: Antiretroviral Therapy
DIC: Drug Information Center
DTC: Dugs Therapeutic Committee
EPSA: Ethiopian Pharmaceuticals Supply Agency
ETB: Ethiopian Birr
HCMIS: Health Commodity Management Information System
HFs: Health Facilities
IPLS: Integrated Pharmaceuticals Logistics System
IV: Intravenous
LMICs: Low and Middle-Income Countries
LMIS: Logistics management information system
MSH: Management Science for Health
OBS and GNY: Obstetrics and gynecology
OPD: Out Patient Department
SNNPR: Southern Nations, Nationalities, and Peoples' Region
TPE: Total Pharmaceutical Expenditure
USAID: United States Agency for International Development

## References

[1] Abdelmonim Ahmed HI, Kheder S, Mohamed Awad M (2019). Pharmaceutical inventory control in Sudan central and hospital stores using ABC-VEN analysis. Global Drugs and Therapeutics..

[2] Wagstaff A, Eozenou P, Smitz M (2020). Out-of-Pocket Expenditures on Health: A Global Stocktake. The World Bank Research Observer.

[3] Castillo CHM, Garrafa V, Cunha T, Hellmann F (2017). Access to health care as a human right in international policy: critical reflections and contemporary challenges. Cien Saude Colet.

[4] World Health Organization. Access to Medicines: making market forces serve the poor. 2007–2017. Geneva, Switzerland: World Health Organization. Accessed 2 Dec 2021. Available from: https://www.who.int/publications/10-year-review/chapter-medicines.pdf?ua=1.

[5] T Pheage. 2017. Dying from lack of medicines;Encouraging local production, right policies the way out. Africa Renewal. Accessed on 5 Dec. 2021. Available from: https://www.un.org/africaAfrica%20Renewalrenewal/magazine/december-2016-march-2017/dying-lack-medicines

[6] Tewuhibo D, Asmamaw G, Ayenew W (2021). Availability of Essential Medicines in Ethiopia: A Systematic Review. Journal of Community Medicine & Health Care.

[7] SefinewMigbaru MY (2016). Berhanemeskel Woldegerima, Workineh Shibeshi ABC-VEN matrix analysis of pharmaceutical inventory management in Tikur Anbessa Specialized Hospital for the years 2009 to 2013, Addis Ababa, Ethiopia. Indian Journal of Basic and Applied Medical Research.

[8] World Health Organization and BILL & MELINDA GATES foundation (2020). Improvement Strategies Model: Drugs & Supplies. Primary Health Care Performance Initiative.

[9] Pund S, Kuril B, Hashmi S, Doibale M, Doifode S (2016). ABC-VED matrix analysis of Government Medical College, Aurangabad drug store. International Journal of Community Medicine and Public Health.

[10] Devarajan D, Jayamohan MS (2016). Stock control in a Chemical Firm: Combined FSN and XYZ Analysis. Procedia Technol.

[11] Gizaw T, Jemal A (2021). How is Information from ABC-VED-FNS Matrix Analysis Used to Improve Operational Efficiency of Pharmaceuticals Inventory Management? A Cross-Sectional Case Analysis. Integr Pharm Res Pract.

[12] Reddy DKVK, Sai DMSS, Prabhu DR (2017). A Study on the Selective Controls of Inventory Management And Application of ABC XYZ Control Matrix in the Cardiology Department of A Tertiary Care Hospital. Journal of Dental and Medical Sciences.

[13] Fitriana I, Gagak Donn R, Cahyo BD (2017). Medicine Inventory Management by ABC-VED Analysis in the Pharmacy Store of Veterinary Hospital, Yogyakarta, Indonesia. Asian Journal of Animal and Veterinary Advances.

[14] Yılmaz F (2018). The drug inventories evaluation of healthcare facilities using ABC and VED analyzes. Istanbul J Pharm.

[15] Krishnaraj B (2016). A Study on ABC-XYZ Analysis in a Pharmacy Store. International Journal of Mathematics and Statistics Invention.

[16] Hashmi AR, Amirah NA, Yusof Y. Organizational performance with disruptive factors and inventory control as a mediator in public healthcare of Punjab, Pakistan. Management Science Letters. 2021;11(1):77–86.

[17] Kefale AT, Shebo HH (2019). Availability of essential medicines and pharmaceutical inventory management practice at health centers of Adama town, Ethiopia. BMC Health Serv Res.

[18] Saedi S, Kundakcioglu OE, Henry AC (2016). Mitigating the impact of drug shortages for a healthcare facility: An inventory management approach. Eur J Oper Res.

[19] Musenbi KP. Drug consumption patterns with clinical and financial implications at Kenyata national hospital: University of Nairobi; 2016. Accessed 15 July 2021. Available from: http://erepository.uonbi.ac.ke/bitstream/handle/11295/100201/Musembi_Drug%20Consumption%20Patterns%20With%20Clinical%20And%20Financial%20Implications%20At%20Kenyatta%20National%20Hospital.pdf?sequence=1&isAllowed=y.

[20] Jobira T, Abuye H, Jemal A, Gudeta T (2021). Evaluation of Pharmaceuticals Inventory Management in Selected Health Facilities of West Arsi Zone, Oromia. Ethiopia Integr Pharm Res Pract.

[21] Mohammed SA, Workneh BD (2020). Critical Analysis of Pharmaceuticals Inventory Management Using the ABC-VEN Matrix in Dessie Referral Hospital. Ethiopia Integr Pharm Res Pract.

[22] Abate SM (2017). Special and Aid Pharmaceuticals ABC-VEN Matrix Analysis of Tikur Anbessa Specialized Hospital for the Years 2009 to 2013, Addis Ababa, Ethiopia. Indian Journal of Basic and Applied Medical Research.

[23] Taddele BW, Wondimagegn AA, Asaro MA, Sorato MM, Gedayi BG, Hailesilase AAJJoYP (2019). ABC-VEN matrix analysis of the pharmacy store in a secondary level health care facility in Arbaminch Town, Southern Ethiopia. J Young Pharm.

[24] Ethiopian Central Statistical Agency (ECA). Summary and Statistical Report of the 2007 Population and Housing Census: Population Size by Age and Sex. Addis Ababa: Federal Democratic Republic of Ethiopia, Population Census Commission; 2008. Accessed 28 July 2021. Available from: https://www.ethiopianreview.com/pdf/001/Cen2007_firstdraft(1).pdf.

[25] Befekadu A, Cheneke W, Kebebe D, Gudeta T (2020). Inventory management performance for laboratory commodities in public hospitals of Jimma zone, Southwest Ethiopia. Journal of pharmaceutical policy and practice.

[26] FMoH. Ethiopian Health Center Reform Implementation Guidelines (EHCRIG). Addis Ababa: Ethiopian Federal Ministry of Health; 2016.

[27] Pharmaceuticals Fund and Supply Agency (2015). Standard Operating Procedures (SOP) Manual for the Integrated Pharmaceuticals Logistics System in Health Facilities of Ethiopia.

[28] Belay H, Azim T, Kassahun H. Assessment of Health Management Information System (HMIS) Performance in SNNPR, Ethiopia. SNNP Regional Health Bureau; 2014. Accessed 23 July 2021. Available from: https://pdf.usaid.gov/pdf_docs/PA00K27K.pdf.

[29] John Snow ID. Logistics Indicators Assessment Tool (LIAT). Arlington: John Snow, Inc./DELIVER, for the U.S. Agency for International Development; 2005. Accessed 11 July 2021. Available from: https://pdf.usaid.gov/pdf_docs/Pnade735.pdf.

[30] Nigah R, Devnani M (2010). Gupta AJJoyp. ABC and VED analysis of the pharmacy store of a tertiary care teaching, research and referral healthcare institute of India.

[31] Mallick B, Dutta ON, Das S. A case study on inventory management using selective control techniques. Journal of the Association of Engineers. 2012;82(1):10–24.

[32] Uthayakumar R, Priyan S. Pharmaceutical supply chain and inventory management strategies: Optimization for a pharmaceutical company and a hospital. Operations Research for Health Care. 2013;2(3):52–64.

[33] Tanwari A, Lakhiar AQ, Shaikh GY. ABC analysis as a inventory control technique. QUEST Research Journal. 2000;1(1).

[34] Ceylan Z, Bulkan S. Drug inventory management of a pharmacy using ABC and VED analysis. Eurasian Journal of Health Technology Assessment. 2017;2(1):14–8.

[35] Ahmed HA, Kheder SI, Awad MM (2019). Pharmaceutical inventory control in Sudan central and hospital stores using ABC-VEN analysis. Glob Drugs Therap.

[36] Mehrotra S., Basukala S. Management of Drugs using 3D Music Inventory Control Technique in a Tertiary Care Hospital. International Journal of Current Research. 2015;7(04):15219–23.

[37] Management Sciences for Health and World Health Organization (2007). Drug and Therapeutics Committee Training Course. Submitted to the U.S. Agency for International Development by the Rational Pharmaceutical Management Plus Program.

[38] Management Sciences for Health (2012). MDS-3: Managing Access to Medicines and Health Technologies.

[39] Brasola L, Di Giorgio D, La Bella F, Pani M, Turchetti GJMAPoC. Medicine thefts and their prevention: current approach in Italy and future perspectives. Research @ Point of Care. 2018;2:1–6. 10.1177/2399202618768676.

[40] Riadi CZ, Hendrawan D, Ambon IM, Pratomo RMH (2020). The ABC-FSN Analysis and Determination of Forecasting Methods for Material Maintenance, Repair & Operations Inventory, Case Study at PT X. Journal of Critical Reviews.

[41] Wagenaar BH, Gimbel S, Hoek R, Pfeiffer J, Michel C, Manuel JL (2014). Stock-outs of essential health products in Mozambique - longitudinal analyses from 2011 to 2013. Trop Med Int Health.

[42] Khembhavi R, Bhojwani K, Bhojwani D, Besekar S (2019). A study to review drug inventory and pharmacy management with reference to I.V. & injectables at a tertiary municipal care hospital with 1800 bedded hospital. The Pharma Innovation Journal..

[43] Manivel P, Ranganathan RJIJP (2016). Prioritized ABC FSN analysis of inventory management in private and hospital pharmacy followed by questionnaire. Int Res J Pharm..

[44] Vrat P. Selective Inventory Management. Materials Management: Springer; 2014:37–49. 10.1007/978-81-322-1970-5

[45] Kassahun TE. The pharmaceutical market in Ethiopia (2015–2018). Ethiopian Investment Commission. 2018. Accessed 8 July 2021. Available from: https://www.unido.org/sites/default/files/files/2018-03/Tilahun%20Kassahun_EIC_%20Market%20Access_02032018%20Bonn.pdf.

